# Connexin hemichannels in the lens

**DOI:** 10.3389/fphys.2014.00020

**Published:** 2014-02-11

**Authors:** Eric C. Beyer, Viviana M. Berthoud

**Affiliations:** Department of Pediatrics, University of ChicagoChicago, IL, USA

**Keywords:** connexin46, connexin50, cataract, lens, gap junction

## Abstract

The normal function and survival of cells in the avascular lens is facilitated by intercellular communication through an extensive network of gap junctions formed predominantly by three connexins (Cx43, Cx46, and Cx50). In expression systems, these connexins can all induce hemichannel currents, but other lens proteins (e.g., pannexin1) can also induce similar currents. Hemichannel currents have been detected in isolated lens fiber cells. These hemichannels may make significant contributions to normal lens physiology and pathophysiology. Studies of some connexin mutants linked to congenital cataracts have implicated hemichannels with aberrant voltage-dependent gating or modulation by divalent cations in disease pathogenesis. Hemichannels may also contribute to age- and disease-related cataracts.

## The lens and cataracts

**Figure 1:**
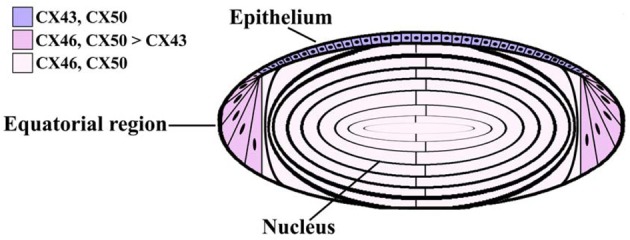
**Diagram of the lens showing the distribution of connexin isoforms**. Cells from the anterior epithelial cell layer express Cx43 and Cx50, differentiating fiber cells express Cx43, Cx46, and Cx50, and fiber cells contain Cx46 and Cx50.

The lens is a transparent organ whose main function is to transmit light and focus it on the retina. It sits suspended between two clear fluids (the aqueous humor and the vitreous) and has no direct blood supply. The lens is comprised of two cell types: epithelial cells that form a single layer along the anterior surface and fiber cells that form the bulk of the organ (Figure 1). At the lens equator, epithelial cells differentiate into fiber cells, a process that involves cell elongation, loss of nuclei and organelles, and synthesis of very high concentrations of small soluble proteins called crystallins. These proteins act as chaperones and increase the refractive index of the lens without interfering with its transparency. This differentiation process, which occurs throughout the lifespan of the organism, leads to generation of two cell types which differ in their metabolic capacities: the surface epithelial cells, which are nucleated and contain most of the metabolic, synthetic, and active transport machinery of the lens and mature fiber cells which have limited metabolic activities, are non-dividing, and must survive for the lifespan of the organism (Mathias and Rae, 2004).

A cataract is an opacity or cloudiness in the lens that may cause a decrease in vision and could eventually lead to blindness. The specific biochemical and structural changes associated with cataract formation are diverse, but a common biochemical change is the generation of high molecular weight insoluble protein aggregates (Moreau and King, 2012).

Because the lens does not have a direct blood supply, the nutrients for the organ all derive from the fluids in which it is suspended. Specifically, the aqueous humor (which is dynamically produced from the plasma) provides the main source for inorganic and organic ions, carbohydrates, glutathione, amino acids, and oxygen. The aqueous humor is also the repository for metabolites and carbon dioxide produced by lens cells. Ions and nutrients reach cells in the interior through an internal “circulation” in which flow of ions and water drives the movement of solutes throughout the organ. A model of this circulation has been developed based on surface currents recorded from lenses (Robinson and Patterson, 1982; Parmelee, 1986; Mathias et al., 1997) and measurements of hydrostatic pressures at different depths within the lens (Gao et al., 2011). In this model, current carried by ions (and associated water and solutes) enters the lens along the extracellular spaces at the anterior and posterior poles, it crosses fiber cell membranes in the lens interior, and it flows back to the surface at the equator (via a cell-to-cell pathway) (Mathias et al., 2007, 2010). The hydrostatic pressure gradient also drives water flow toward the exterior (Gao et al., 2011). The lens circulatory system provides a pathway for internal fiber cells to obtain essential nutrients, remove potentially toxic metabolites, and maintain resting potentials (Goodenough, 1979; Piatigorsky, 1980).

## Lens gap junctions and connexins

Intercellular communication among the cells of the lens is facilitated by an extensive network of gap junctions. Gap junctions are membrane specializations that contain clusters of intercellular channels that are permeable to ions and small solutes (≤1 kDa). Epithelial and fiber cells contain morphologically and physiologically distinct gap junctions (Rae and Kuszak, 1983; Miller and Goodenough, 1986). Epithelial cells are functionally coupled through gap junction channels. Lens fiber cells also share ions and small metabolites through gap junction channels, and consequently behave as a functional syncytium (Goodenough et al., 1980; Mathias and Rae, 1989). The extent of epithelial-to-fiber cell coupling is somewhat controversial.

Gap junction channels are oligomeric assemblies of members of a family of related proteins called connexins (Cx) (Beyer and Berthoud, 2009). Six connexins oligomerize to form a connexon (hemichannel), and two connexons dock to form a complete (dodecameric) intercellular channel. Channels formed by diverse connexins differ in physiological properties including unitary conductance, permeability, gating, and regulation by different protein kinase-dependent pathways (reviewed in Harris, 2001, 2007; Sáez et al., 2003). Thus, the regulation of intercellular communication and the permeation of different molecules in different regions of the lens are determined by the repertoire of connexins expressed.

Three connexins have been identified in the lens with somewhat overlapping expression patterns (Figure 1). Cx43 is expressed in lens epithelial cells (Musil et al., 1990), but its expression is turned off as epithelial cells in the equatorial region differentiate into fiber cells. Cx50 is also expressed in epithelial cells (TenBroek et al., 1994; Dahm et al., 1999; Rong et al., 2002). Cx46 and Cx50 become abundantly expressed in the differentiating cells and are the two most abundant connexins in lens fiber cells (Paul et al., 1991; White et al., 1992). These two connexins co-localize at gap junction plaques and can form mixed hexamers (Paul et al., 1991; Jiang and Goodenough, 1996). Transcripts for a fourth connexin, Cx23, have been detected in the zebrafish embryo lens (Iovine et al., 2008) and in the mouse lens (Puk et al., 2008). This connexin has been implicated in fiber cell differentiation, because fiber cells do not elongate properly in mice expressing a missense mutation of Cx23 (Puk et al., 2008). While lens cells from other mammalian species may also express Cx23, Cx23 transcript was not detected in RNA isolated from human lenses (Sonntag et al., 2009). Moreover, while Cx23 protein has been detected in proteomic studies of mouse lens membrane proteins (Bassnett et al., 2009), it was not detected in human samples (Wang et al., 2013). Even in the mouse, the cellular distribution of the Cx23 protein is unknown, because there are no good antibodies for its detection. Therefore, Cx23 is not included in Figure 1.

## Studies of Cx46- or Cx50-null mice implicate connexins in cataract formation

The importance of gap junction-mediated lens intercellular communication for the maintenance of lens transparency has been substantiated by a number of genetic studies in mice. Targeted deletion of either Cx46 or Cx50 results in the development of cataracts in homozygous (but not heterozygous) null mice (Gong et al., 1997; White et al., 1998). The Cx50-null mice have a milder cataract than the Cx46-null mice (Gerido et al., 2003). Cx50-null mice have microphthalmia and small lenses, while eye and lens sizes are similar in Cx46-null and wild type mice (Gong et al., 1997; White et al., 1998; Rong et al., 2002). Double knock-out mice lacking both Cx46 and Cx50 have small lenses with dense opacities that are far more extensive than those observed in either Cx46 or Cx50 single null mice (Xia et al., 2006). Cx43 may not have a critical importance in normal lens function, because the lenses of animals with a conditional deletion of Cx43 are transparent and develop normally through at least 6 months of age, even though intercellular transfer of neurobiotin and Lucifer yellow among epithelial cells is decreased (DeRosa et al., 2009).

## Lens connexin mutations are linked to congenital cataracts

Mutations in lens connexins have been linked to disease in people and rodents. Missense and frame-shift mutations of the genes encoding Cx46 and Cx50 (*GJA3* and *GJA8*) have been identified in members of human families with inherited cataracts of various different phenotypes. Nearly all of the cataracts are inherited as autosomal dominant traits. These mutants and their associated cataract phenotypes have been reviewed recently (Beyer et al., 2013). In several mutant mouse strains, the cataract trait has also been mapped to mutations of Cx46 and Cx50.

The functional and cellular abnormalities associated with cataract-linked connexin mutants have been thoroughly studied for some of the mutants. The most frequently observed phenotype is induction of no or insignificant levels of intercellular conductance and formation of no or very few gap junction plaques. Examples include Cx50R23T, Cx50D47N, Cx50P88S, Cx50P88Q, and Cx46fs380 (Berthoud et al., 2003; Minogue et al., 2005; Arora et al., 2006, 2008; Thomas et al., 2008). All of the mutants with this general phenotype (loss of function and a severe reduction in the number or complete absence of gap junctions) should reduce intercellular communication between lens fiber cells, regardless of the differences in the mechanisms for their trafficking impairment, retention, or accumulation.

Other mutants (e.g., Cx50W45S, Cx46D3Y, and Cx46L11S) make abundant gap junction plaques, but have no gap junction channel activity when expressed by themselves implying that they form non-functional channels (Tong et al., 2011, 2013). They have an open probability of zero (or no unitary conductance).

A connexin mutant may also contribute to cataractogenesis through interactions with the co-expressed wild type connexins. In the lens, expression of a mutant connexin may affect the abundance/stability of the wild type connexins. For instance, in both heterozygous and homozygous lenses of *No2* mice, expression of Cx50D47A leads to severe reductions in the levels of both Cx50 and Cx46 (likely by increasing degradation of the wild type and mutant connexins) (Berthoud et al., 2013). Intercellular communication between fiber cells of *No2* mouse lenses is likely severely reduced. A mutant connexin may also affect the function of the wild type connexins. Exogenous expression experiments show that some mutants (e.g., Cx50P88S, Cx50P88Q, Cx50W45S, Cx50E48K, Cx46D3Y, Cx46L11S) decrease the junctional conductance supported by their wild type counterparts, likely through co-oligomerization (Pal et al., 1999; Arora et al., 2006; Banks et al., 2009; Tong et al., 2011, 2013).

Several different mechanisms have been invoked to explain how changes in intercellular communication lead to cataract formation. Reductions of intercellular communication should decrease the circulation of gap junction permeant molecules (including water, ions, and metabolites) between lens cells. Consistent with that prediction, Cx46- and Cx50-null mice have reduced coupling conductances between lens fibers (reviewed in Mathias et al., 2010) and a decrease in the gradient of intracellular hydrostatic pressure that normally runs from the center to the periphery of the lens (Gao et al., 2011). Data from Cx46-null mice suggest that impairment of lens intercellular communication leads to increased levels of intracellular calcium within the lens which contribute to cataract formation by stimulating calpain-dependent proteolysis of crystallins (Baruch et al., 2001; Gao et al., 2004).

In the lens, mutant connexins may also contribute to cataracts by altering the trafficking or function of other (non-connexin) lens fiber cell proteins. The mutant connexins may also interfere with lens development and differentiation and, consequently, alter the expression of these other lens proteins.

## Connexin hemichannels

In addition to forming intercellular channels, connexins can form functional hemichannels that induce large conductances in single plasma membranes. These conductances are caused by permeation of ions through “undocked” single connexons. This phenomenon has been best demonstrated in expression systems and in cultures of various cells that endogenously express connexins.

**Figure 2:**
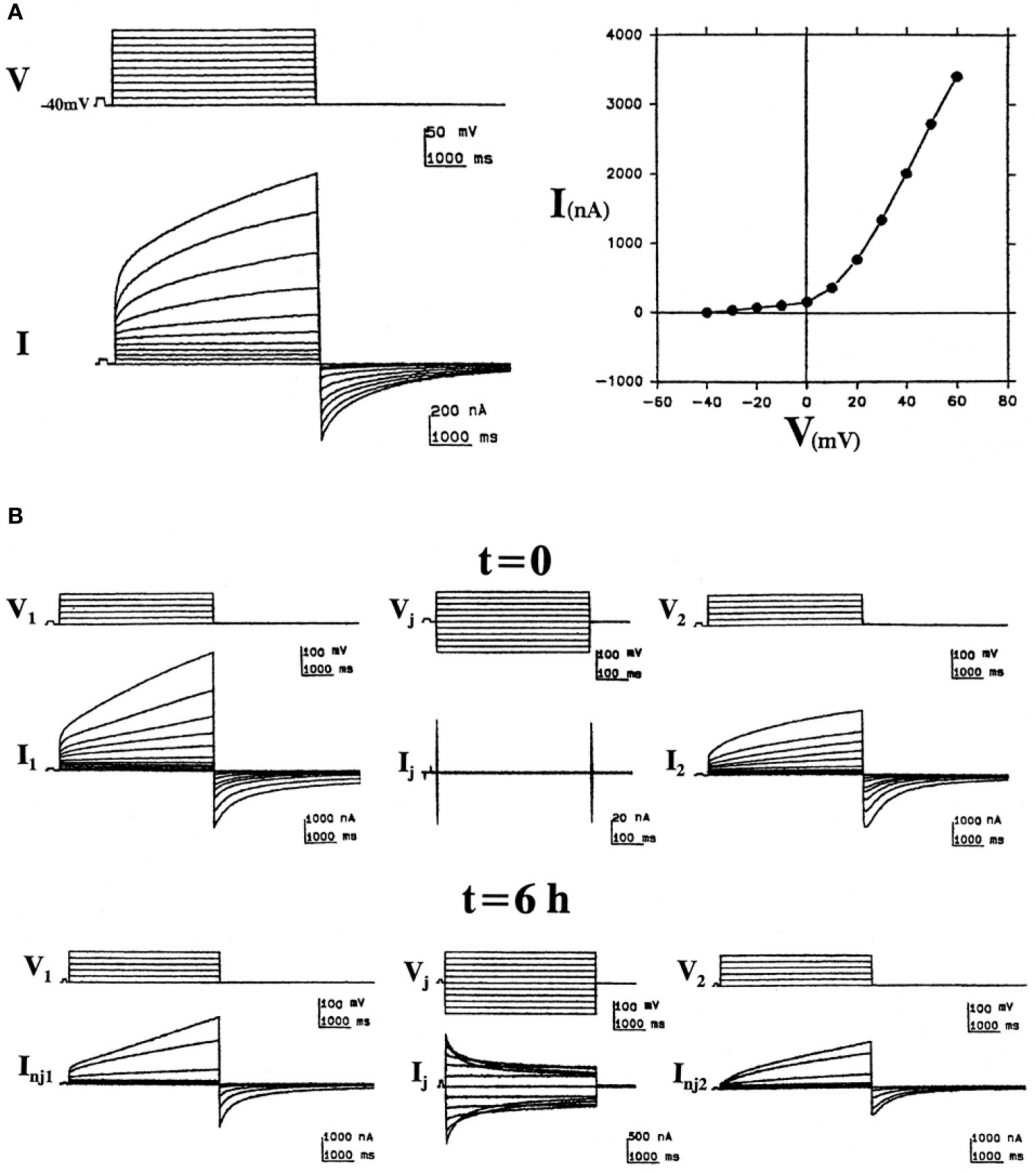
**“Hemichannel” currents induced by expression of the bovine Cx46 ortholog (Cx44) in *Xenopus* oocytes. (A)** Bovine Cx44-induced non-junctional currents in isolated oocytes. Left: Sample recordings illustrate the induction of an outward time- and voltage-dependent current (*I*) activated by depolarizing voltage pulses (*V*) of 5 s from a holding potential of −40 mV to +60 mV in increments of 10 mV. At pulse-off, the currents deactivated returning to baseline. Right: The corresponding *I*/*V* curve, plotting the current values at the end of the pulses vs. voltage. **(B)** Formation of junctional channels from bovine Cx44 hemichannels. Left and right panels show non-junctional currents of bovine Cx44-injected oocytes (1 and 2) at the time of pairing (*t* = 0) and 6 h after pairing (*t* = 6 h). The middle panels show the junctional currents at these times (reproduced from Gupta et al., 1994).

Cx46 was the first connexin demonstrated to form hemichannels in *Xenopus* oocytes and has been one of the most studied. After cloning rat Cx46 cDNA, Paul et al. (1991) injected the *in vitro* transcribed cRNA into oocytes, and they were surprised to observe depolarization and osmotic lysis. Voltage-clamp experiments revealed that Cx46 cRNA-injected oocytes developed a large, non-selective cation current that was activated on depolarization and was inhibited by external divalent cations (Ebihara and Steiner, 1993). Subsequent studies showed that while Cx46 hemichannels allow permeation of both cations and anions, they are more permeable to cations (Trexler et al., 1996). Examples of the hemichannel currents induced by expression of the bovine Cx46 ortholog (Cx44) are shown in Figure 2A. By recording both non-junctional and junctional currents from the time of pairing, Gupta et al. (1994) showed that hemichannels could be recruited into intercellular channels (reproduced in Figure 2B). Cx46 hemichannels are gated closed by low intracellular pH; this closure is voltage dependent (Trexler et al., 1999). The single channel conductance of Cx46 hemichannels exhibits significant rectification; it is 300 pS at −50 mV, but only 135 pS at +50 mV (Trexler et al., 1996).

Like Cx46, all of the other lens connexins (including Cx43, Cx50, and Cx23) can form functional hemichannels (Beahm and Hall, 2002; Contreras et al., 2003; Srinivas et al., 2005; Sonntag et al., 2009). However, detection of comparable levels of hemichannel current may require injection of larger amounts of RNA for Cx43 and Cx50 than for Cx46 (Tong and Ebihara, 2006). The hemichannels formed by each of these connexins are regulated by voltage and extracellular divalent cations; hyperpolarization and elevated divalent cation concentrations promote closure. Synergistic action of these two mechanisms may prevent the opening of hemichannels under physiological conditions. Among the lens connexins, only Cx46 has a weak enough Ca^2+^-sensitivity that it may exhibit significant opening at physiological concentrations of extracellular Ca^2+^. Quantification of the differences in divalent cation sensitivity has shown that hemichannel currents induced by chicken Cx45.6 (the ortholog of mammalian Cx50) can only be detected when the external calcium concentration is reduced to zero nominal concentration, whereas Cx46 hemichannel currents (and those of its chicken ortholog, Cx56) are detectable at much higher external calcium concentrations (0.7 mM Ca^2+^) (Ebihara et al., 1995). It is not completely clear what determines the difference in Ca^2+^ sensitivity between these connexins, nor the mechanism by which Ca^2+^ promotes hemichannel closure. In the case of Cx46 hemichannels, Verselis and Srinivas (2008) have shown that calcium only closes Cx46 hemichannels in excised patches when added from the extracellular side. In the case of Cx50, Zhang et al. have proposed that Ca^2+^ regulates Cx50 hemichannels by influencing calmodulin binding to the cytoplasmic side (Zhang et al., 2006), similar to the Ca^2+^/calmodulin regulation of other connexin intercellular channels (Peracchia et al., 2000; Zhou et al., 2009; Xu et al., 2012). Observations following application of a thiol reactive compound to rat Cx46 containing a cysteine substitution for leucine35 imply that Ca^2+^ gates the channel closed at a position that is extracellular to leucine35 (Pfahnl and Dahl, 1999).

Expression studies have shown that the lens connexins form hemichannels that also differ in some other properties. Their hemichannel currents exhibit differences in activation and deactivation kinetics (Ebihara et al., 1995). They have unique single channel conductances. They differ in their permeabilities to some small molecules.

When expressed in HeLa cells or in *Xenopus* oocytes, Cx50 forms high conductance (352 or 470 pS) single hemichannels (Valiunas and Weingart, 2000; Srinivas et al., 2005). Hemichannels formed of Cx50 are sensitive to extracellular monovalent cations. Replacement of extracellular Na^+^ with K^+^ may reduce the ability of Ca^2+^ (or other divalent cations) to close Cx50 hemichannels (Srinivas et al., 2006).

Cx43 hemichannels have largely been described and studied in non-lens cell types such as astrocytes. They have unitary conductances of ~220 pS (about twice the conductance of a single Cx43 intercellular channel) (Contreras et al., 2003). In addition to opening provoked by low concentrations of extracellular divalent cations, Cx43 hemichannels open in response to various cellular insults like metabolic inhibition, ischemia, and lowering of the intracellular redox potential (Contreras et al., 2002, 2004; Retamal et al., 2006, 2007). Some of these opening events are associated with S-nitrosylation of Cx43 (Retamal et al., 2006). Cx43 hemichannels are permeable to a variety of common dye tracers (like Lucifer yellow, ethidium, and propidium) and can allow the release of cytoplasmic small molecules (including ATP and glutamate) (Orellana et al., 2011). Cx23 is a unique member of the connexin family. Unlike other connexins, it only contains four (instead of six) cysteines in its extracellular loops. When studied in stably transfected HeLa cells, mouse Cx23 supported release of ATP (even in calcium containing medium), but did not induce detectable intercellular currents nor it allow passage of microinjected tracers (Sonntag et al., 2009). These observations suggested that mouse Cx23 formed hemichannels, but not gap junction channels. In contrast, in another study, the Cx23 orthologs from zebrafish formed both gap junction channels and hemichannels (Iovine et al., 2008). Likely because its expression cannot be detected in primates (including humans) (Sonntag et al., 2009), there have been very few studies of Cx23 and its hemichannels.

There have been some studies of the pharmacology of hemichannels formed by the lens connexins. These hemichannels are generally inhibited by similar concentrations of the non-selective blockers that inhibit most gap junctional channels (including carbenoxolone, α-glycyrrhetinic acid, flufenamic acid, heptanol, and octanol). Cx46 hemichannels are modulated by PKC-dependent phosphorylation. Treatment with the PKC activators, phorbol-12-myristate-13-acetate (TPA) or 1-oleoyl-2-acetyl-*sn*-glycerol (OAG), reduces the amplitude of hemichannel currents and leads to their inactivation after prolonged incubation, an effect that can be reverted by PKC inhibitors (Ngezahayo et al., 1998; Jedamzik et al., 2000). Treatment with a casein kinase II inhibitor (2-dimethylamino-4,5,6,7-tetrabromo-1H-benzimidazole) during the expression period also reduced the amplitude of the currents evoked by a 60 mV depolarizing pulse (Walter et al., 2008). Cx46 hemichannels are also sensitive to nitric oxide donors (e.g., *S*-nitrosoglutathione); Cx46 hemichannel currents show a faster activation rate, increased voltage sensitivity, and increased tail currents with altered kinetics in single *Xenopus* oocytes incubated in the presence of *S*-nitrosoglutathione. These effects have been ascribed to nitrosylation of cytoplasmic cysteines, because they were not observed when these cysteines were mutated to alanines (Retamal et al., 2009). Unsaturated fatty acids can also modulate Cx46 hemichannel function. Linoleic acid has a biphasic effect on Cx46, increasing hemichannel currents at 0.1 μM and decreasing them at concentrations of 100 μM or higher (without affecting gap junction channels) (Retamal et al., 2011).

Like Cx50 gap junction channels, Cx50 hemichannels are inhibited by mefloquine and other quinine derivatives (like N-benzylquininium) (Cruikshank et al., 2004; Rubinos et al., 2012). Cx43 channels and hemichannels are also inhibited by this drug, but much higher concentrations are required. Cx46 is virtually insensitive.

In expression systems, some of the lens connexins can form functional heteromeric hemichannels with characteristics that differ from those of either connexin alone. Co-expression of chicken Cx56 (orthologous to mammalian Cx46) with Cx50 (or its chicken ortholog, Cx45.6) produced hemichannels with intermediate properties in several parameters including the threshold for activation, rate of deactivation, unitary conductance, steady state open probability, and mean open times at negative potentials (Ebihara et al., 1999).

Because Cx46 and Cx50 form hemichannels in expression systems, they have been extensively analyzed after mutagenesis in structure-function studies. Sequential replacement of individual amino acid residues with cysteines in Cx46 and determination of their accessibility to modifying reagents has implicated residues in the first transmembrane and first extracellular domains in formation of the channel pore (Zhou et al., 1997; Kronengold et al., 2003). Other studies have implicated the first extracellular domain of Cx46 in determining charge selectivity (Trexler et al., 2000). Reciprocal substitution experiments have implicated N-terminal amino acids as critical determinants of the differences in voltage gating properties between Cx46 and Cx50 (Tong and Ebihara, 2006). Truncations that remove portions of the C-terminus of Cx46 have implicated this domain in hemichannel function (Zeilinger et al., 2005; Walter et al., 2008). In some studies, these truncation mutants induce hemichannel currents of smaller magnitude than those of the full length wild type protein, which may result from decreased trafficking to/from the plasma membrane (Schlingmann et al., 2013).

## Connexin hemichannels in the lens

Several lines of evidence demonstrate the presence of “hemichannel-like” activities in the lens. Their sensitivities to calcium and to some pharmacologic inhibitors suggest that they may be formed by connexins. When Rae et al. (1992) removed extracellular divalent cations from whole lenses, they observed a decrease in resting membrane potential and a large increase in input conductance, which they ascribed to activation of a stretch activated, non-selective cation channel. Eckert et al. (1998) identified a large, slowly-activating, non-selective current in lens fiber cells isolated under calcium-free conditions. Hyposmotic stress induced ATP release and uptake of propidium in intact lenses that were blocked by 18α-glycyrrhetinic acid and probenecid (Shahidullah et al., 2011, 2012).

**Figure 3:**
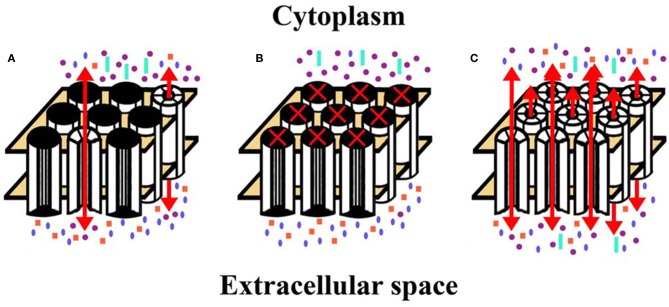
**Diagrams represent hemichannel function in the lens in normal physiology and in pathology. (A)** Under normal conditions, few connexin hemichannels are open, permitting only a small flux of ions according to their concentration gradients. This would mostly include influx of Na^+^ and Ca^2+^ and efflux of K^+^. **(B)** Reduction (or complete blockade) of hemichannel opening would reduce the normal, physiologic transmembrane passage of permeant ions and solutes leading to alterations of the normal ionic concentrations across the plasma membrane. **(C)** If hemichannel opening was pathologically increased, ions and other small molecules would flow across the membrane according to their concentration gradients. Movement of electrolytes like Na^+^ and K^+^ would lead to loss of transmembrane potentials. Entry of Ca^2+^ might lead to opening of additional hemichannels, and activation of several signaling cascades and calcium-dependent proteases. A significant increase in the intracellular Ca^2+^ concentration may lead to cell death by different mechanisms (Orrenius et al., 2003). Increased hemichannel opening would also lead to loss of ATP, NAD^+^, glutathione, and other permeant cytoplasmic small molecules. All of these changes would contribute to loss of homeostasis and cytotoxicity. Na^+^, blue ellipses; K^+^, purple circles; Ca^2+^, orange squares; ATP (or NAD^+^, glutathione, etc.), aquamarine rectangles.

Ebihara et al. (2010) further examined hemichannel currents in isolated lens fiber cells by whole cell patch clamping. Upon removal of divalent cations from the external solution, they detected large, non-selective currents that activated on depolarization. A single channel conductance of 241–243 pS was measured in a few cells. Uptake of propidium iodide and 4'-6-diamidino-2-phenylindole (DAPI) was observed in divalent cation-free medium, and the dye uptake was inhibited by gap junction blockers including Gd^3+^, flufenamic acid, and octanol. The calcium-sensitive currents and dye uptake were detected in fiber cells isolated from Cx50-null mice, but they were absent in cells isolated from Cx46/Cx50 double-null mice. These data suggest that Cx46 hemichannels are responsible for the calcium-sensitive currents and dye uptake, and that Cx46 hemichannels may be functional in the lens *in vivo*. Opening of hemichannels in the lens may have significant physiological roles (Figure 3A). Because Cx46 hemichannels are mechanosensitive, it has been proposed that their openings allow rapid fluid equilibration during lens accommodation (Bao et al., 2004b). Ebihara et al. (2010) have suggested that connexin hemichannels may provide a normal pathway for influx of calcium and sodium in fiber cells. Although Cx46 hemichannels have a low probability of opening under normal conditions (i.e., at membrane resting potentials and 1 mM [Ca^2+^]_o_), they might be sufficient to account for the rather low sodium influx that occurs under physiological conditions in fiber cells.

## Other lens channels that resemble connexin hemichannels

There are also other proteins that form channels with properties similar to connexin hemichannels and may contribute to lens physiology and pathophysiology.

The pannexins are a family of proteins with three members in mouse and man (Panx1, Panx2, and Panx3). They were originally discovered based on their sequence similarity to the innexins which form gap junctions in invertebrates (Panchin et al., 2000). Although most expression studies indicate that pannexins do not form intercellular channels, there is agreement that they form large pore, non-selective transmembrane channels with some similarities and differences as compared with connexin hemichannels. The properties of pannexin channels, their roles in various different cell types and comparisons to connexin hemichannels have been extensively reviewed (MacVicar and Thompson, 2010; Sosinsky et al., 2011).

Among the pannexins, Panx1 has been most extensively studied. Panx1 channels can open in the presence of physiological extracellular calcium concentrations. They are mechanosensitive, and they can be activated by high extracellular K^+^ (Bao et al., 2004a; Pelegrin and Surprenant, 2006; Silverman et al., 2009). Panx1 channels allow uptake of various dye tracers (Bao et al., 2004a; Pelegrin and Surprenant, 2006; Silverman et al., 2009) and release of ATP from erythrocytes, taste receptors, and other cells (Locovei et al., 2006; Huang et al., 2007). Panx1 associates with the P2X_7_ purinergic receptor and contributes to formation of the inflammasome (Pelegrin and Surprenant, 2006; Silverman et al., 2009). Panx1 channels are blocked by some of the same agents as connexins (like carbenoxolone, flufenamic acid, and mefloquine) but also by some others (like probenecid) that do not block connexin channels (Iglesias et al., 2008; Ma et al., 2009).

There is rather limited information regarding pannexins in the lens. Both Panx1 and Panx2 mRNAs are expressed in the lens, and immunoreactive Panx1 is detected in lens epithelial and fiber cells (Dvoriantchikova et al., 2006). It has been hypothesized that Panx1 forms the probenecid-inhibitable channels that release ATP from lens epithelial cells after exposure to hyposmotic stress (Shahidullah et al., 2011, 2012). Gunning et al. (2012) have found that lens fiber cells contain a non-specific cation conductance that may be distinct from connexin hemichannels. It is stimulated by hypertonic stress or isosmotic cell shrinkage and may be involved in volume regulation. Members of the transient receptor potential (TRP) family of cation channels are also expressed in the lens, and activation of TRPV4 channels may participate in the osmotic stress-stimulated release of ATP from the lens (Shahidullah et al., 2011, 2012). However, connexin hemichannels could also contribute to this process, since Cx43 hemichannels have been implicated in regulation of cell volume in other cells (Quist et al., 2000).

## Connexin hemichannels and cataracts

Studies of cataract-linked connexin mutants suggest several ways that connexin hemichannels contribute to the pathogenesis of cataracts (Figures 3B,C).

The ability of some mutant lens connexins to form functional hemichannels has been assessed. Unlike wild type Cx46, many of the cataract-associated Cx46 mutants do not form functional hemichannels (e.g., Cx46L11S, Cx46fs380). Others exhibit a reduced ability to form them (e.g., Cx46D3Y, Cx46N63S) (Pal et al., 2000; Tong et al., 2013). These mutants would lead to less than normal connexin hemichannel activity in the lens (Figure 3B).

Cataract-associated mutants may also form hemichannels with altered properties (e.g., gating or charge selectivity) as compared with the wild type connexin. For example, the cataract-associated mutant Cx46N63S (which does not form functional gap junction channels) is impaired in its ability to induce hemichannel currents under standard recording conditions (i.e., 0.7 mM [Ca^2+^]_o_ and 0.8 mM [Mg^2+^]_o_), but Cx46N63S-induced currents increase in magnitude when the concentration of the divalent cations is decreased (Ebihara et al., 2003). This mutant forms hemichannels with increased sensitivity to the extracellular concentration of magnesium ions (Ebihara et al., 2003). Cx46D3Y forms hemichannels that have altered charge selectivity and voltage-dependent gating (Tong et al., 2013).

**Figure 4:**
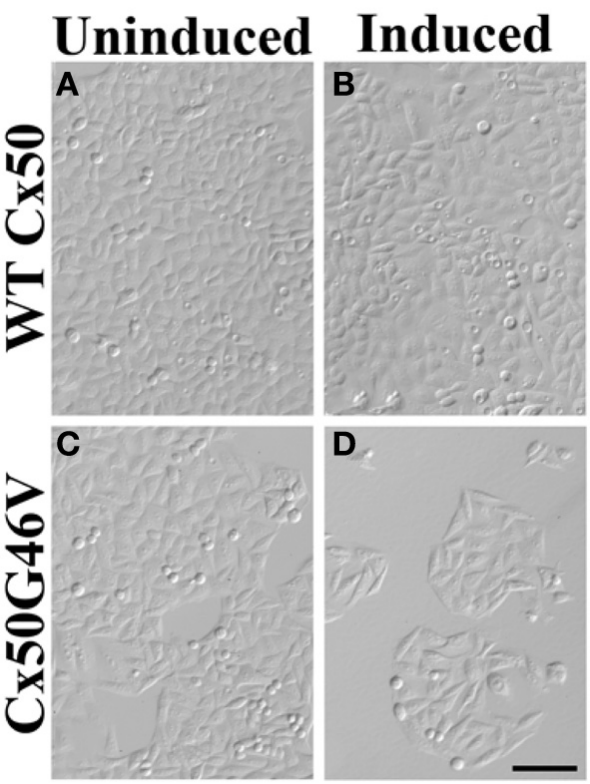
**Expression of Cx50G46V (but not wild type Cx50) decreased the number of cells**. HeLa cells were stably transfected with constructs that allowed the inducible expression of wild type (WT) Cx50 or Cx50G46V. Phase-contrast photomicrographs obtained from cultures of cells transfected with WT Cx50 **(A,B)** or with Cx50G46V **(C,D)** that were left untreated **(A,C)** or that were induced by treatment with 1 μM ponasterone A **(B,D)** for 96 h. While a dramatically reduced number of cells remained in the culture 96 h after induction of Cx50G46V, the cell density was not affected by induction of wild type Cx50. This reduction is consistent with the cytotoxicity anticipated for this mutant due to its increased hemichannel function. Bar, 111 μm (reproduced from Minogue et al., 2009 with minor modifications).

A very striking alteration of hemichannel properties is exemplified by Cx50G46V, a mutant found in a patient with total cataract (Minogue et al., 2009). This mutant forms gap junction plaques and supports intercellular communication normally. However, unlike wild type Cx50, Cx50G46V has a greatly increased ability to form functional hemichannels (Minogue et al., 2009; Tong et al., 2011). Expression of this mutant increases the proportion of apoptotic cells and causes cell death (Minogue et al., 2009) (Figure 4), suggesting that opening of the hemichannels would also cause severe cell damage *in vivo*. This cytotoxicity appears dominant, since co-expression of Cx50G46V with wild type Cx46 or Cx50 also decreases cell (oocyte) viability (Tong et al., 2011). Recently, a cataract-linked human Cx46 mutant (Cx46G143R) was identified that had increased hemichannel function, but no gap junction channel function (Ren et al., 2013). Connexin mutants with enhanced hemichannel activity (Figure 3C) may cause fiber cell death through a complex sequence of events including disruption of transmembrane ion gradients leading to loss of membrane potential, and entry of calcium ions, leading to activation of intracellular proteases and decreased metabolic activity.

## Possible contributions of connexin hemichannels to acquired cataracts

The roles of connexin hemichannels in age- or disease-related cataracts are unknown and largely unexplored. However, there are a variety of reasons to hypothesize that alterations of the lens connexins and their hemichannel activities might contribute to the development of these cataracts.

Connexin hemichannels may facilitate the disturbances of calcium homeostasis that contribute to the pathogenesis of various different kinds of cataracts. The hemichannels can be activated by depolarization, which is frequently associated with the onset of cataractogenesis and allow entry of Ca^2+^ from the extracellular space. Studies in isolated lens fiber cells have shown that influx of calcium ions can provoke a process termed “disintegrative globulization” that may mimic cataractogenesis (Bhatnagar et al., 1995). This process can also be induced by osmotic changes or hyperglycemia as may occur in association with diabetes (Wang et al., 1997; Chandra et al., 2002). Although chloride channels and L-type calcium channels have been implicated in this process (Wang et al., 1996, 1997), it is reasonable to hypothesize that lens connexin hemichannels might also participate as conduits for entry of calcium ions (Ebihara et al., 2010).

Aberrant hemichannel opening may also be involved in cataracts that are associated with various stress factors. Age-related cataracts are thought to result from the cumulative effects of oxidative stress on lens components (i.e., DNA, proteins, and lipids) and the decreased efficiency of repair mechanisms. In astrocytes, the metabolic inhibition-induced opening of Cx43 hemichannels was associated with S-nitrosylation of the protein and blocked by high intracellular concentrations of reduced glutathione (Retamal et al., 2006). In the normal lens, the high concentrations of glutathione that are present may similarly keep connexin hemichannels closed and protect the lens from their potential deleterious consequences. However, in cataractous lenses where levels of glutathione are decreased (Truscott and Augusteyn, 1977), increased hemichannel opening might lead to further deterioration of the organ. However, the regulation of connexin hemichannel opening by reducing agents may depend on the cell type and the initial state of the cell. Studies of connexin constructs expressed by retroviral infection of chicken embryo fibroblasts have suggested that ultraviolet radiation stimulates caspase-dependent cleavage of Cx50 which leads to closure of hemichannels and reduction of intercellular communication (Wang et al., 2012). These authors speculate that this might be a mechanism for the lens to protect itself against this cataract-causing radiation.

On the other hand, studies have also suggested that some of the cellular components that contribute to lens pathology might reduce the normal physiologic opening of hemichannels. Such alterations would disrupt normal Cx46 hemichannel functions including serving as conduits for the entry of sodium and calcium ions into lens cells. Indeed, unsaturated fatty acids like linoleic acid block Cx46 hemichannels at the concentrations found in the lens and thus might contribute to cataract formation (Retamal et al., 2011).

## Conclusions and perspectives

It is established that the lens connexins can make functional hemichannels in exogenous expression systems and in lens cells and that a connexin mutant that causes aberrant opening of hemichannels causes cataracts. Future studies should expand and clarify our understanding of the roles of connexin hemichannels in the physiology of the normal lens and in the pathogenesis of cataracts due to many different causes.

A low level of hemichannel function may contribute to the normal physiology of the lens (as illustrated in Figure 3A). The best evidence implicates Cx46 in the connexin hemichannel openings detected in isolated lens cells (Ebihara et al., 2010). However, it is likely that Cx43 and Cx50 also contribute to the hemichannels present in the lens and that heteromeric hemichannels are formed among the three connexins.

It will be important to answer several questions regarding the roles of connexin hemichannels in normal lens physiology. How is the opening of these hemichannels regulated in the lens? Are connexin hemichannels responsible for the chronic Na^+^ current in the normal lens? Do connexin hemichannels participate in the volume regulation accompanying accommodation? Do they participate in the lens circulation of water and solutes?

Reduced opening of connexin hemichannels (Figure 3B) should disrupt all of these normal processes. It might affect accommodation or even lead to cataracts.

Conversely, increased hemichannel opening (Figure 3C) might also lead to lens pathology. In the rare connexin mutant exhibiting gain of hemichannel function, the mechanism of cataract formation needs to be clarified. It can be anticipated that it involves disruption of transmembrane ion gradients and loss of cytoplasmic components (like ATP). In addition, it is possible that abnormal opening of connexin hemichannels contributes to the pathogenesis of cataracts that have a high incidence due to non-genetic etiologies. The disease mechanisms for such cataracts include activation of kinases, imbalances of redox potentials, and accumulation of calcium which might increase hemichannel opening following pathophysiologic modification of the connexins by phosphorylation, oxidative damage, etc.

Direct testing of these hypotheses would be facilitated by development of selective pharmacologic inhibitors of the connexin hemichannels or by genetic approaches like generation of mutant mice. For example, a mouse could be generated by replacing the wild type gene encoding Cx46 or Cx50 with a mutant that only makes functional intercellular channels (but not hemichannels). In these mice, experiments could evaluate whether the mice develop cataracts (either normally or in response to stresses like ultraviolet radiation or diabetes) and whether the fiber cells have normal resting potentials and ionic currents. A positive outcome of such studies might imply the potential therapeutic value of a hemichannel-inhibiting drug as a treatment to prevent development of cataracts.

### Conflict of interest statement

The authors declare that the research was conducted in the absence of any commercial or financial relationships that could be construed as a potential conflict of interest.
